# Integrated transcriptomic and metabolomic analysis of flavonoid biosynthesis in cigar tobacco leaves under variable nitrogen regimes

**DOI:** 10.3389/fpls.2025.1589215

**Published:** 2025-06-23

**Authors:** Kai Jia, Juanjuan Shi, Lingli Bai, Xueren Wang, Yuemin Wang, Xiangzhen Li, Wenqing Li, Chaoyuan Zheng

**Affiliations:** ^1^ College of Resources and Environment, Fujian Agriculture and Forestry University, Fuzhou, Fujian, China; ^2^ Sanming Tobacco Sciences Institute, Sanming Tobacco Company of Fujian Province, Sanming, Fujian, China; ^3^ Institute of Tobacco Sciences, Fujian Provincial Tobacco Monopoly Bureau, Fuzhou, Fujian, China

**Keywords:** nitrogen, transcriptomics, metabolomics, flavonoids, cigar tobacco

## Abstract

**Introduction:**

Flavonoids are the most abundant secondary metabolites in plants and play important roles in plant growth, environmental adaptation, and human health. Tobacco serves as a vital cash crop and key source of flavonoids. Given tobacco's high nitrogen demand and the particular sensitivity of flavonoid synthesis to nitrogen supply, understanding the regulatory mechanisms of flavonoid biosynthesis under varying nitrogen conditions is essential.

**Methods and results:**

We systematically investigated the flavonoid biosynthesis pathways in the cigar tobacco cultivar Haiyan 204 under different nitrogen application rates using integrated transcriptomic and metabolomic analyses. This study identified 694 differentially expressed metabolites (DEMs) and 6,114 differentially expressed genes (DEGs). Integrated analyses revealed significant enrichment in the flavonoid biosynthesis and phenylpropanoid biosynthesis pathways. A regulatory network of flavonoid biosynthesis in response to nitrogen was constructed. Weighted gene co-expression network analysis (WGCNA) identified CHS2 as a key gene strongly associated with flavonoid biosynthesis, alongside its potential regulatory transcription factors *MYB7*, *MYB9*, *bHLH14*, and *MYB_related1*. The reliability of transcriptome data was validated via qPCR.

**Discussion:**

These results provide a scientific basis for elucidating the biosynthesis mechanisms of tobacco flavonoids and identifying key nitrogen-responsive genes. The constructed regulatory network and identified transcriptional regulators offer critical insights into nitrogen-modulated flavonoid synthesis in cigar tobacco.

## 1 Introduction

Flavonoids are among the most prevalent secondary metabolites in plants and are frequently present in fruits, vegetables and other crops, mainly concentrated in flowers, leaves and seeds. Flavonoids regulate plant growth and development and are integral to plant adaptation to biotic and abiotic stresses (Shen et al., 2022; Shi et al., 2021). As antioxidants or detoxifiers in plants, they have the ability to eliminate reactive oxygen species (ROS), which effectively protects plants from biotic and abiotic stresses such as UV radiation, cold stress, pathogen infection and insect nibbling (Cavaiuolo et al., 2013; Iwashina, 2003; Pourcel et al., 2007; Zhang et al., 2020). Meanwhile, flavonoids also have beneficial biochemical effects on human beings, helping to delay aging and prevent various diseases (Fernandes et al., 2017; Imran et al., 2019; Selvakumar et al., 2020). Flavonoid biosynthesis initiates from the phenylpropanoid pathway and branches into diverse secondary metabolites, including flavonoids, flavonols, isoflavones and anthocyanins, through intermediates such as coumaroyl coenzyme A and chalcone (Dong and Lin, 2021; Liu et al., 2021a; Nabavi et al., 2020). The biosynthesis of flavonoids is jointly regulated by internal factors such as structural genes, transcription factors, MiRNAs and environmental factors such as temperature, light, water, nutrition and hormones, among which transcription factors play a key role in the synthesis process by regulating the expression of structural genes (Liu et al., 2021b; Xu et al., 2015; Ye et al., 2019). The biosynthesis of flavonoids is regulated by a variety of enzymes, including but not limited to: PAL (phenylalanine ammonia-lyase), C4H (cinnamate 4-hydroxylase), 4CL (4-coumarate-CoA ligase), CHS (chalcone synthase), CHI (chalcone isomerase), F3H (flavanone 3-hydroxylase), FLS (flavonol synthase), ANS (anthocyanidin synthase) etc (Liu et al., 2021a). Song’s research on the flavonoid biosynthesis mechanism in camellia seeds found that this process is regulated by transcription factors such as MYC2, bHLH3, bHLH18, etc (Song et al., 2022). In *Sophora alopecuroides*, flavonoid biosynthesis-related genes are primarily regulated by transcription factors such as MYB, bHLH, and NAC (Zhu et al., 2021). Additionally, the flavonoid biosynthesis in tea plants is also regulated by MYB, bHLH, and WD transcription factors (Wang et al., 2018; Zhao et al., 2013). These findings indicate that the genes and regulatory factors related to flavonoid biosynthesis vary among different plants depending on species, gene families, functions, and experimental setups, and other factors.

Nitrogen is an important nutrient element necessary for the biosynthesis of amino acids and secondary metabolites and a key factor limiting crop yield (Li et al., 2017; Maathuis, 2009). Studies have shown that appropriate nitrogen application rates can improve the agronomic traits of tobacco, increase the accumulation of dry matter in cigar tobacco leaves, and enhance its economic value (Chen et al., 2020; Yang et al., 2022). As carbon-based secondary metabolites, the biosynthesis of flavonoids is strongly influenced by plant nitrogen status (Deng et al., 2019; Liu et al., 2010b). Research has shown that while fertilization increases the yield of medicinal plants, vegetables, and crops, it may also reduce the accumulation and synthesis of active compounds such as flavonoids and terpenes, thereby affecting their quality (Groenbaek et al., 2016; Tshivhandekano et al., 2013). It has also been shown that nitrogen fertilization can reduce the accumulation of flavonoids in the leaves of *Gynostemma pentaphyllum*, and increased nitrogen nutrition also leads to a decrease in flavonoid content in apple leaves (Cavaiuolo et al., 2013; Leser and Treutter, 2005; Rühmann et al., 2002). Adequate nitrogen levels promote flavonol glycoside biosynthesis through gene regulation and the accumulation of substrate carbohydrates, while imbalanced nitrogen, especially excess nitrogen, can suppress this process (Dong et al., 2019).

Tobacco (*Nicotiana tabacum L.*) belongs to the Solanaceae family, as a distinct tobacco product, the primary difference between cigars and cigarettes is that cigars are wrapped in tobacco leaves, whereas cigarettes are wrapped in paper (Yang et al., 2022). In addition, tobacco has a high medicinal value and is an important source of medicinal alkaloids and flavonoids, especially rutin, the content of which is closely related to the quality, aroma and flavor of the tobacco leaf (Fathi et al., 2006; Gebhardt, 2016; Williamson and Gwynn, 1982). Although flavonoid biosynthetic pathways have been extensively studied, flavonoid synthesis is specific, and the biosynthetic processes and expression levels of related genes vary depending on plant species, developmental stage and tissue type (Wan et al., 2020). No studies have been seen on the flavonoid biosynthetic pathways in cigar tobacco leaves, especially in response to nitrogen application rates. The aim of this study was to elucidate the effect of nitrogen on flavonoid biosynthetic pathways in cigar tobacco leaves through transcriptomics and metabolomics approaches. This information will provide an important reference for developing optimal cultivation strategies to improve the quality of cigar tobacco leaves.

## 2 Materials and methods

### 2.1 Plant materials and treatment

The field trial was conducted in Sanyuan District, Sanming City (E117°16’-117°48’, N26°01’-26°25’), a region with a mid-subtropical monsoon climate. The site experiences an annual precipitation of 1,500–1,900 mm, of which over 70% occurs during the cigar tobacco growing season (January to June). The mean annual temperature is 19.4°C, with an average sunshine duration of 1,394.7 hours during the primary growth period and an annual frost-free period ranging from 248 to 352 days (mean 305 days).

The cigar core variety Haiyan 204 was selected for this study. A total of five nitrogen fertilizer gradients (according to the amount of pure nitrogen application rates) were set as follows: N0 (0 kg N/hm²), N1 (120 kg/hm²), N2 (180 kg/hm²), N3 (210 kg/hm²), and N4 (240 kg/hm²). The fertilizers for each treatment were prepared in specific proportions using Ca(NO_3_)_2_·4H_2_O, KNO_3_, NH_4_HCO_3_, K_2_SO_4_, KH_2_PO_4_, Mg(OH)_2_, and calcium-magnesium-phosphate. The preparation principle ensured that, except for the differing nitrogen amounts, the phosphorus and potassium application rates remained consistent across treatments. Calcium, magnesium, and phosphate were mixed with fine soil and applied as hole fertilizers; NH_4_HCO_3_ along with some KNO_3_ and K_2_SO_4_ were used as top-dressing; the remaining fertilizers were applied as base fertilizers in furrows, N:P_2_O_5_:K_2_O=1:1:2.64. The basic soil properties are as follows: pH 5.75, organic matter 1.23%, total nitrogen 0.072%, available phosphorus 1.44 mg/kg, and available potassium 164.38 mg/kg.

The experiment was conducted in a randomized block design initiated on 21 January 2022. Each treatment comprised 60 plants per plot with three replications, and row spacing was maintained at 120 cm × 35 cm. Samples were taken on 21 June, when the upper leaves in the field were at maturity, on the 86th day after transplanting. Each treatment consisted of three replicates, so that we obtained samples from fifteen plots (2 to 4 tobacco leaves per plot) based on five N application treatments. The leaf veins were removed and frozen in liquid nitrogen and brought back to the laboratory. Based on this samples were milled in liquid nitrogen for phenotypic data and subsequent transcriptomics and metabolomics assays.

### 2.2 Chlorophyll, total free amino, superoxide dismutase, total flavonoid, lignin determination

Leaf samples were flash-frozen in liquid nitrogen, lyophilized, and homogenized to a fine powder for subsequent assays. Chlorophyll a and b concentrations were quantified spectrophotometrically at 663 nm and 645 nm, respectively (Porra et al., 1989). Free amino acid extraction followed the ninhydrin reaction protocol (Moore and Stein, 1954), while total flavonoids were assessed via the NaNO_2_–Al(NO_3_)_3_–NaOH colorimetric assay (Lu et al., 2021). Superoxide dismutase(SOD) activity was measured by nitroblue tetrazolium photoreduction (Elavarthi and Martin, 2010), and lignin content was analyzed through the Klason gravimetric procedure (Lupoi et al., 2015).

### 2.3 Determination of agronomic traits figures and phenotypic data analysis

Agronomic traits of tobacco plants were measured 1 week after topping, including plant height, stem circumference, pitch, and number of leaves. Measure with a tape measure. Phenotypic data were analyzed using one-way ANOVA with multiple comparisons conducted using Duncan’s method.

### 2.4 Transcriptome assay

#### 2.4.1 RNA extraction, cDNA library construction and sequencing

Using the same sample as 2.1, a total of fifteen (five treatments, each repeated three times), the RNA purification, reverse transcription, library construction, and sequencing were carried out at Shanghai Majorbio Bio-pharm Biotechnology Co., Ltd. (Shanghai, China). Total RNA was extracted using the MJZol Total RNA Extraction Kit (Shanghai Meiji Junhua Biomedical Technology Co., Ltd., China). The RNA concentration and purity were determined using a NanoDrop 2000 (Thermo Fisher Scientific, USA). RNA integrity was evaluated through agarose gel electrophoresis using Biowest Agarose (Biowest, Spain), and the RIN value was obtained using an Agilent 5300 (Agilent Technologies, USA). For library construction, the RNA requirements included a total amount of 1 μg, a concentration of ≥ 30 μg/μL, an RIN > 6.5, and an OD260/280 ratio between 1.8 and 2.2. The library was constructed using the Illumina NovaSeq Reagent Kit method (Illumina, USA)to obtain the final library.

#### 2.4.2 Transcriptome data processing and functional annotation of genes

Reference gene source: *Nicotiana_tabacum*; Reference genome version: GCF_000715135.1(https://www.ncbi.nlm.nih.gov/genome/?term=txid4097[orgn]). In the transcriptome data, raw paired-end reads were trimmed and quality controlled by fastp v0.19.5 (Chen et al., 2018a) with default parameters. Reads containing adapters, those without insert fragments, and those with ambiguous bases (N, more than five) were removed. Low-quality bases at the start and end of the sequences were trimmed, and sequences shorter than 30 bp after adapter removal and quality trimming were discarded. The clean reads were then aligned to the reference genome with orientation mode using HISAT2 v2.1.0 (Kim et al., 2015). The mapped reads of each sample were assembled using StringTie v2.1.2 (Pertea et al., 2015) in a reference-based approach. Gene expression levels in RNA-Seq analysis were determined by quantifying the number of sequences mapped to genomic regions.

#### 2.4.3 Statistical analysis of gene data

The expression level of each transcript was calculated using the transcripts per million reads (TPM) method to identify DEGs between two samples. RSEM software (v1.3.3, University of Wisconsin-Madison, USA) (Li and Dewey, 2011) was used to quantitatively analyze the expression levels of genes and transcripts, enabling the subsequent differential expression analysis between different samples. By combining functional information of the sequences, the regulatory mechanisms of the genes can be revealed. The software used for differential expression analysis is DESeq2 (v1.24.0, Bioconductor, USA) (Love et al., 2014), with the criteria for significant differential expression set at *p* < 0.05 & |log2FC|≥1. Statistical significance was set at *p* < 0.05 and results are expressed as mean ± standard error. Bar graphs were used to show up and down-regulated DEGs between different groups. Comparative analysis of different groups of differential genes using Venn diagrams. Enrichment analyses of major pathways were performed using the KEGG database. Cluster analysis was performed using the R (v4.3.0) package: fastcluster (Müllner, 2013), and gene expression levels (TPM) were clustered after logarithmic transformation (log10), and subclusters were illustrated.

### 2.5 Metabolite analysis

#### 2.5.1 Metabolite extraction

Using the same sample as 2.1, a total of fifteen (five treatments, each repeated three times) First, accurately weigh 100 ± 5 mg of the sample into a 2 mL centrifuge tube and add a 6 mm diameter grinding bead. Add 400 µL of extraction solvent (Methanol: water=4:1), which includes four internal standards (L-2-chlorophenylalanine at 0.02 mg/mL, e.g.). Use the Wonbio-96C freeze tissue grinder (Shanghai Wanbo Biotechnology Co., Ltd., China) to grind for 6 minutes (-10°C, 50 Hz). Use the SBL-10TD temperature-controlled ultrasonic cleaning machine (Ningbo Xinzhi Biotechnology Co., Ltd., China) for low-temperature ultrasonic extraction for 30 minutes (5°C, 40 KHz), allow the samples to sit at -20°C for 30 minutes. Then, centrifuge using the Centrifuge 5430R (Eppendorf, Germany) at 13,000 g for 15 minutes (13000 g, 4°C) and transfer the supernatant to sampling vials with inserts for analysis.

#### 2.5.2 Metabolite detection and analysis of chromatographic conditions

Use the Thermo Fisher Scientific Ultra High Performance Liquid Chromatography Tandem Fourier Transform Mass Spectrometry (UHPLC-Q Exactive) system (Thermo Fisher Scientific, USA) for LC-MS analysis (Shanghai Majorbio Bio-pharm Biotechnology Co., Ltd.). The chromatography conditions involve using an ACQUITY UPLC BEH C18 (100 mm*2.1 mm i.d, 1.7 µm; Waters, Milford, USA); The mobile phase A consists of 2% acetonitrile in water (containing 0.1% formic acid), while mobile phase B is acetonitrile (containing 0.1% formic acid). The injection volume is 3 μL, and the column temperature is maintained at 40 °C. Mass spectrometry conditions: Samples are ionized using electrospray ionization (ESI) and mass spectral signals are collected in both positive and negative ion scanning modes. The scanning mode is set to full scan (SCAN), with a mass scanning range of m/z 50-500. The ion spray voltage is set to 3500 V for positive ion mode and -3000 V for negative ion mode. Normalized collision energy is optimized for the detection of metabolites.

#### 2.5.3 Metabolome data preprocessing and metabolite identification

Using Progenesis QI (Waters Corporation, Milford, USA) for peak extraction, alignment, identification, etc., a data matrix is generated containing retention time, peak area, mass-to-charge ratio, and identification information for further processing and bioinformatics analysis. Afterwards, the software was used for feature peak search library identification, matching MS and MS/MS mass spectrometry information with the self-built plant specific metabolite database (MJDBPM). The MS mass error is set to less than 10 ppm, and metabolites are identified based on the scores from the MS/MS matching.

#### 2.5.4 Statistical analysis of metabolome data

Significant differential metabolites are selected based on the variable importance in projection (VIP) values obtained from the OPLS-DA model and the results of Student’s t-test, with criteria set at p < 0.05 & |log2FC|≥1 & VIP > 1.0. The identified DEMs are annotated for metabolic pathways using the KEGG database (https://www.kegg.jp/ke-gg/pathway.html) to determine the pathways in which these metabolites are involved. Metabolomic data analysis involved sample correlation analysis and principal component analysis (PCA) to validate biological replicates and assess similarities among different treatment samples. Bar plots were utilized to display up-regulated and down-regulated differential metabolites across different groups, complemented by Venn diagrams for comparative analysis. The KEGG database was referenced to illustrate secondary metabolic pathways associated with the differential metabolites. Clustering analysis was conducted using the R (v4.3.0) package: fastcluster (Müllner, 2013), with relative expression levels of metabolites log-transformed (log10) for clustering purposes.

### 2.6 Combined transcriptome and metabolome analysis

A joint analysis was conducted on pathways commonly annotated by DEGs and DEMs to display the enriched metabolic pathways across different groups. Each group was selected to show ten enriched pathways, based on the *p* value of DEGs and DEMs as well as the number of associated metabolites. Notably, both DEGs and DEMs were significantly enriched in the plant Flavonoid biosynthesis pathway (pathway KO00941), according to the Kyoto Encyclopedia of Genes and Genomes (KEGG). Weighted gene co-expression network analysis was performed using the R package(v4.3.0): WGCNA (Langfelder and Horvath, 2008) to construct gene co-expression networks. All genes were grouped into different modules based on their expression patterns, with genes within the same module exhibiting strong correlations and similar expression profiles. The correlation and significance between the gene matrix values and core modules of differentially accumulated metabolites were calculated using significance function in the R WGCNA package Relationships between transcription factors, core genes and metabolites were examined using Pearson correlation analysis and visualized using Cytoscape v3.3.0 (Cytoscape Consortium, San Diego, CA, USA).

### 2.7 qPCR verification of flavonoid biosynthesis-related key genes

Total RNA was extracted using the MJZol Total RNA Extraction Kit. The RNA purification, reverse transcription, library construction, and sequencing were carried out at Shanghai Majorbio Bio-pharm Biotechnology Co., Ltd. (Shanghai, China). qPCR was performed using the CFX Connect™ real-time PCR system (Bio-Rad, USA) with 2 × ChamQ Universal SYBR qPCR Master Mix (Vazyme, China) and MagicSYBR Mixture (CW3008, CWBIO, China). Actin was used as the internal reference gene. Each reaction included three biological and technical replicates to ensure accuracy. The amplification conditions were as follows: 95°C for 10 min (pre-denaturation); 40 cycles of 95°C for 10 s (denaturation), 60°C for 30 s (annealing), and 72°C for 30 s (extension). The melt curve analysis was conducted using the instrument’s default settings. Gene expression levels were calculated using the 2^(-ΔΔCt) method (Livak and Schmittgen, 2001). The primers employed in this study are listed in **Supplementary Table S1**.

## 3 Results

### 3.1 Changes of tobacco leaves after nitrogen application rates

Generally, nitrogen application significantly increased the biomass of tobacco plants, therefore, preliminary agronomic traits were determined in this study (**Supplementary Table S2**). Nitrogen fertilizer treatments significantly increased the height, stem circumference and spacing, and number of tobacco leaves.

Phenotypic data and plant condition at maturity in the field (**Supplementary Figure S1**). Significant changes were also observed in the color of the tobacco leaves, which were lighter in the N0 treatment as compared to the nitrogen application treatment. Preliminary analysis of the tobacco leaves showed a significant increase in both total free amino acid and chlorophyll content with higher N application. Amino acid synthesis and photosynthetic capacity in tobacco leaves were significantly increased by nitrogen application. The activity of SOD decreased significantly with increasing nitrogen application, while nitrogen also significantly reduced the total flavonoid content of the tobacco leaves.

### 3.2 Differential gene transcriptome analysis

Through high-throughput sequencing, each sample obtained between 68,678,358 ~ 80,461,874 raw reads. After trimming adapters and filtering out low-quality sequences, each sample produced between 68,021,938 ~ 79,599,370 clean reads. The distribution of Q30 bases in the clean reads exceeded 94.15%, with an average GC content of 42.97% (**Supplementary Table S3**). The alignment rate of the transcriptome data to the reference genome ranged from 93.04% to 95.47% (**Supplementary Table S4**). A total of 69,782 genes were obtained.

**Figure 1:**
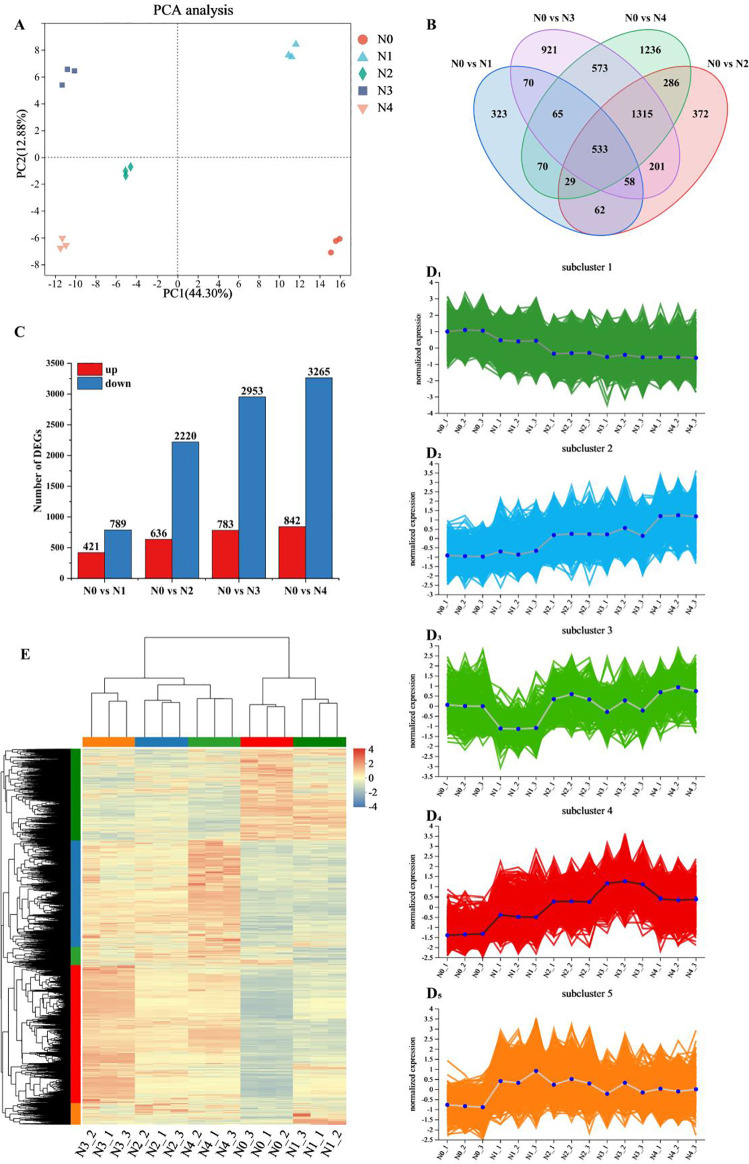
Transcriptome data analysis of tobacco leaves after different nitrogen treatments. **(A)** PCA analysis of gene expression levels. **(B)** Venn diagram of common or unique expression of DEGs between the groups. **(C)** DEGs quantity statistics. **(D_1-5_)** Hierarchical clustering of five sub clusters. **(E)** Cluster analysis of DEGs.

Principal component analysis of the gene expression levels of the 15 samples (**Figure 1A**) revealed that the differences between the two principal components were significant, accounting for 44.3% and 12.88% of the total variance, respectively. The biological replicates were closely clustered, while samples from different treatments were distinctly separated, indicating significant differences in the transcriptome results of tobacco leaves after nitrogen treatment. However, the N0 and N1 treatments were different compared to the other treatments. There were 1,210, 2,856, 3,736 and 4,107 differentially expressed genes in the comparisons of N0 *vs* N1, N0 *vs* N2, N0 *vs* N3 and N0 *vs* N4, respectively, of which 421, 636, 783 and 842 were up-regulated and 789, 2,220, 2,953 and 3,265 were down-regulated. The number of DEGs exhibited a dose-dependent increase in response to elevated N supply, and the comparison between N0 *vs* N4 showed the highest number of up-regulated and down-regulated genes (**Figure 1B**). Venn analysis identified 533 genes co-expressed across all N treatment comparisons, highlighting conserved transcriptional responses (**Figure 1C**) and the exclusive differential genes increased with increasing nitrogen application, these results indicated that nitrogen application had a significant effect on these differential genes.

A total of 6,114 DEGs were identified by screening for significant with *p* < 0.05 and |log2FC|≥1. Hierarchical cluster analysis (**Figure 1E**) was performed on this gene set. The results showed significant differences between samples from different treatments, which was consistent with the results of PCA analysis. DEGs from the N0 and N1 treatments showed high similarity, which distinguished them from the other treatments, and most of the DEGs showed an increasing trend with increasing N application. Hierarchical clustering results classified all DEGs into five groups, and all samples showed good reproducibility (**Figure 1D**_1-5_). Subcluster 1 contained 1,499 DEGs, which showed a decreasing trend with increasing nitrogen content. Subcluster 2 contained 1,725 DEGs, and these DEGs tended to increase with the increase of nitrogen fertilizer application. Subcluster 3 had 297 DEGs, subcluster 5 had 342 DEGs, and subcluster 4 contained 2,251 DEGs, which generally increased with increasing nitrogen application, with the highest expression levels observed in nitrogen treatment 3. Overall, subclusters 1, 2 and 4 showed significant changes in relation to the level of nitrogen application.

### 3.3 Differential metabolite analysis

Through metabolomic analysis, a total of 694 DEMs were obtained. The screening criteria for differential metabolites were set at *p* < 0.05 & |log2FC|≥1 & VIP > 1.0. Among the identified metabolites, 40 were flavonoid compounds (**Supplementary Table S4**). These includes 2 types of anthocyanins, 1 type of flavanol, 2 types of flavanones, 5 types of flavones, 20 types of flavonoid glycosides, 3 types of flavonols, and 7 types of isoflavones.

**Figure 2:**
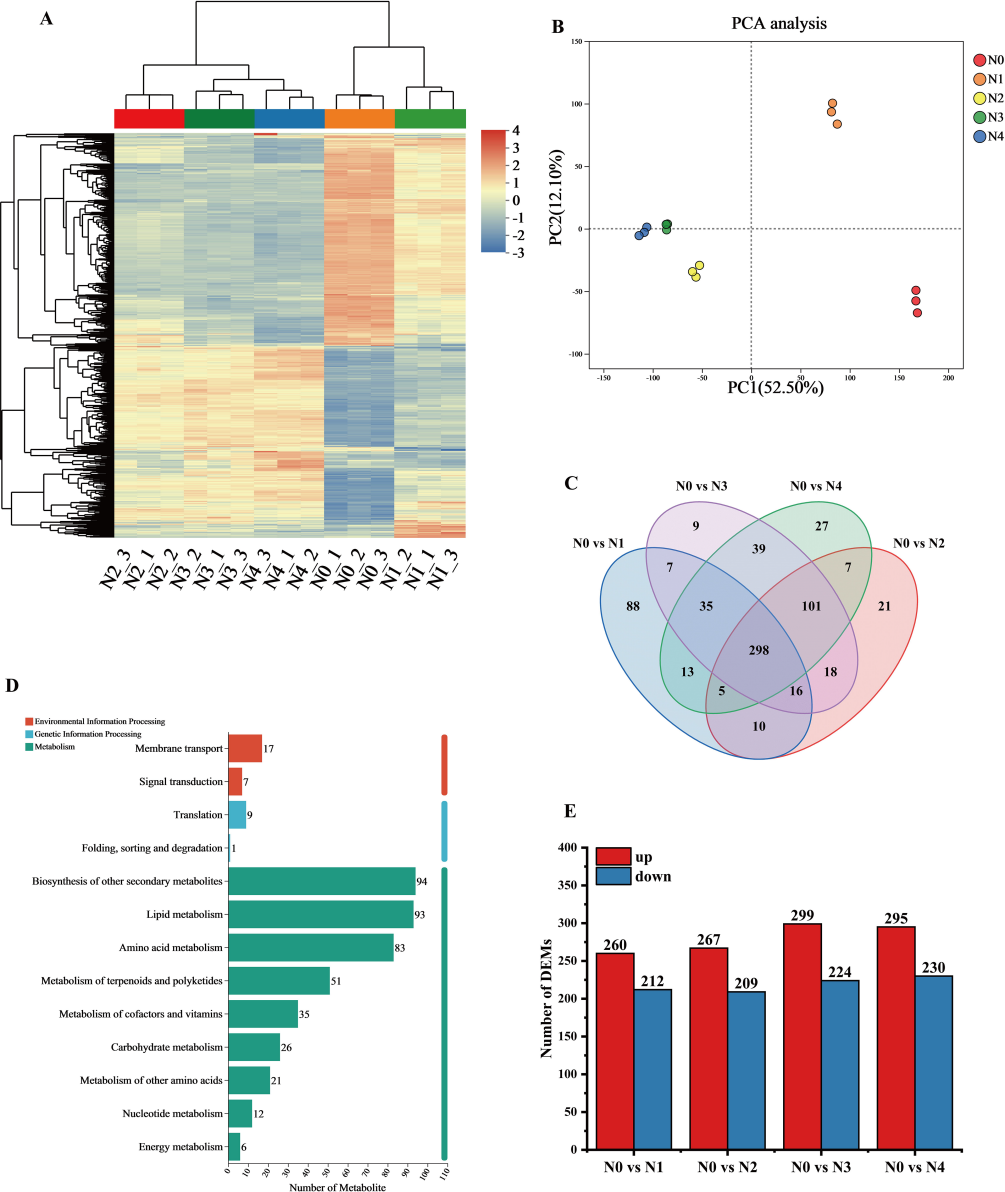
Metabolomics data analysis of tobacco leaves after different nitrogen treatments. **(A)** Clustering analysis of DEMs. **(B)** PCA analysis of metabolite expression levels; **(C)** Venn diagram showing common or unique expression of DEMs between the groups; **(D)** KEGG pathway annotation of metabolites; **(E)** Statistical summary of the number of differential DEMs.

Hierarchical cluster analysis of the metabolite sets showed significant differences among the samples from different treatments. the DEMs between N0 and N1 showed high similarity, which distinguished them from the other treatments, and most of the DEMs showed an increasing trend with increasing N application (**Figure 2A**). Principal component analysis of the metabolites of the 15 samples (**Figure 2B**) showed that the two principal components accounted for 52.5% and 12.1% of the total variance, respectively, which were significantly different. The samples showed proximity within replicates, whereas significant separation was observed between treatments. the N0 and N1 treatments were significantly different from the other treatments, suggesting significant changes in tobacco leaf metabolites after N application.

Venn analysis showed (**Figure 2C**) that there are 298 common differential metabolites shared among the four comparison groups. In contrast to genes, the unique metabolites are most abundant in the N0 *vs* N1 group and least abundant in the N0 *vs* N3 group. Based on KEGG functional annotation, the DEMs were grouped into three categories, with the majority mapped to metabolic pathways: 94 metabolites were annotated to “Biosynthesis of other secondary metabolites” 93 metabolites were annotated to “Lipid metabolism” and 83 metabolites were annotated to “Amino acid metabolism” (**Figure 2D**). Most differential metabolites were up-regulated in N0 and N1 treatments, while downregulation was more prevalent in N3 and N4 treatments (**Figure 2E**). Specifically, the number of genes in the comparisons of N0 *vs* N1, N0 *vs* N2, N0 *vs* N3, and N0 *vs* N4 were 476, 2856, 3736, and 4107, respectively. Overall, nitrogen application significantly influenced the synthesis of metabolic products.

### 3.4 Flavonoid biosynthesis pathway map

**Figure 3:**
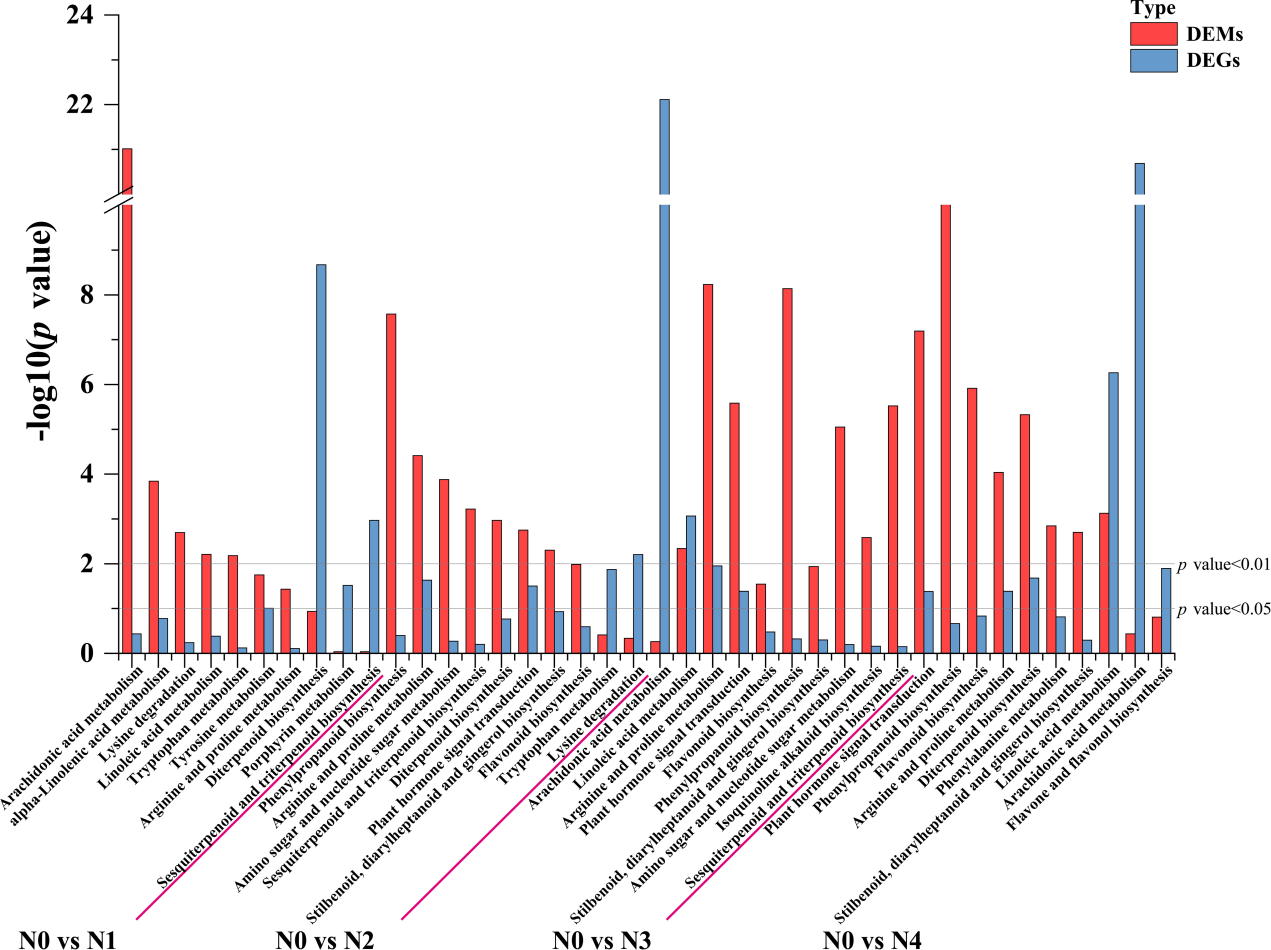
Statistical chart of the top 10 KEGG pathways jointly enriched by DEGs and DEMs in each control group after different nitrogen treatment.

**Figure 4:**
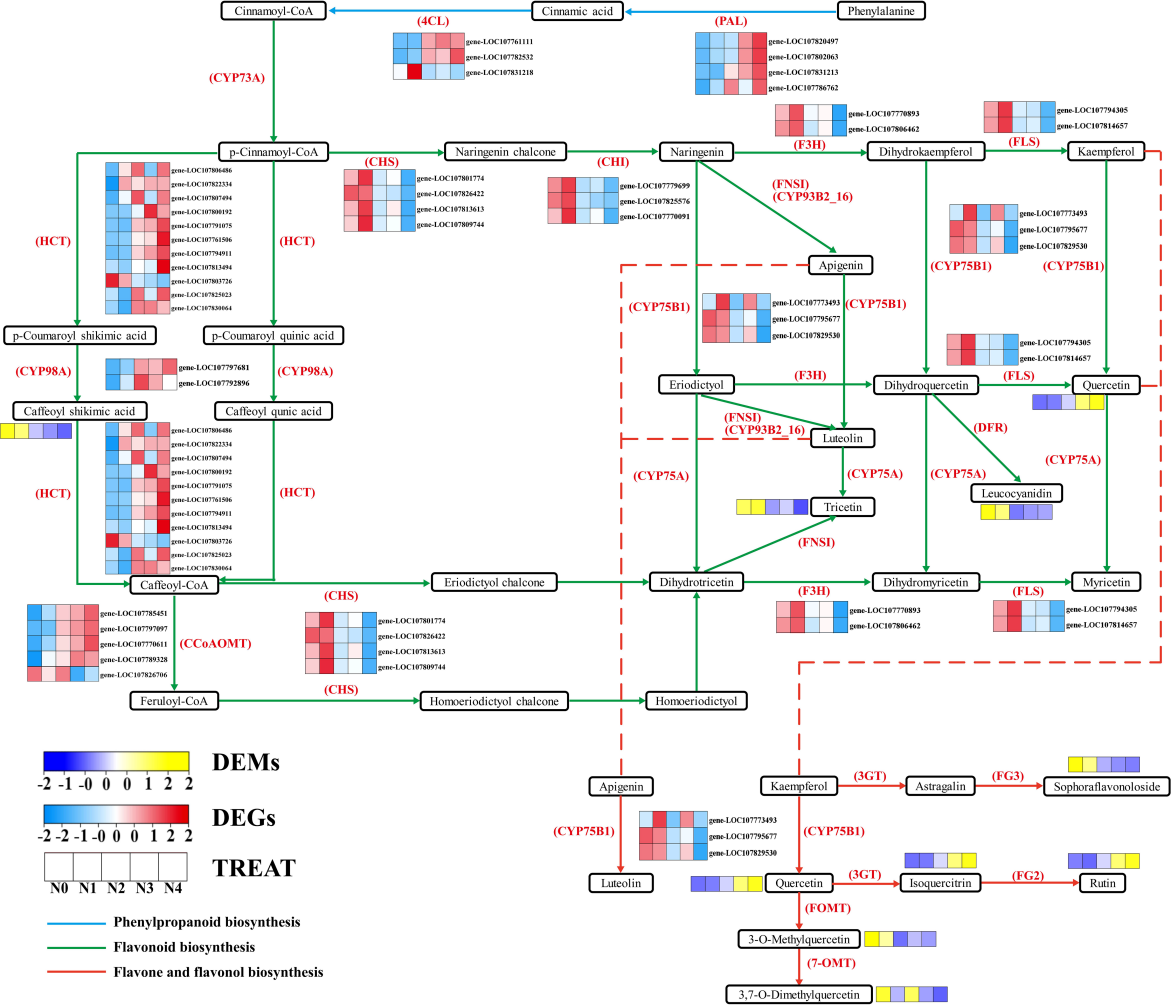
Flavonoid biosynthesis pathway.

To further determine the metabolic pathways co-enriched with DEGs and DEMs, KEGG pathways co-enriched with DEGs and DEMs in each comparison group were used. By combining the *p* value of DEGs and DEMs along with the number of metabolite and gene, 10 enriched pathways were selected for each group (**Supplementary Table S6**). The metabolic pathways co-annotated across different comparison groups showed a high degree of overlap, with “Arginine and proline metabolism” appearing four times, and pathways such as “Flavonoid biosynthesis”, “Diterpenoid biosynthesis”, “Phenylpropanoid biosynthesis”, “Linoleic acid metabolism”, “Arachidonic acid metabolism” appearing three times. Nitrogen treatment significantly influenced the synthesis of amino acids, flavonoids, terpenes, and lipids in tobacco leaves (**Figure 3**). Flavonoids are produced from phenylalanine through the phenylpropanoid pathway, and our results indicate that nitrogen treatment greatly affects pathways involved in flavonoid biosynthesis (Phenylpropanoid biosynthesis and Flavonoid biosynthesis). To clearly and intuitively display the variations in genes and metabolites in the flavonoid biosynthesis pathway of cigar tobacco leaves after nitrogen treatment, a pathway map was created (**Figure 4**). This pathway primarily consists of three routes: Phenylpropanoid biosynthesis, Flavonoid biosynthesis, and Flavone and flavonol biosynthesis.

Through KEGG enrichment analysis and gene functional annotation, a total of 39 genes and 10 corresponding enzymes were identified (**Supplementary Table S7**). The identified enzymes include: PAL (phenylalanine ammonia-lyase [EC:4.3.1.24]), 4CL (4-coumarate-CoA ligase [EC:6.2.1.12]), CYP73A (trans-cinnamate 4-monooxygenase [EC:1.14.14.91]), HCT (shikimate O-hydroxycinnamoyltransferase [EC:2.3.1.133]), CCoAOMT (caffeoyl-CoA O-methyltransferase [EC:2.1.1.104]), CYP98A (5-O-(4-coumaroyl)-D-quin-ate 3’-monooxygenase [EC:1.14.14.96]), CHI (chalcone isomerase [EC:5.5.1.6]), CHS (chalcone synthase [EC:2.3.1.74]), F3H (naringenin 3-dioxygenase [EC:1.14.11.9]), CYP75B1(flavonoid 3’-monooxygenase [EC:1.14.1-4.82]]), FLS (flavonol synthase [EC:1.14.20.6]). Additionally, there are 9 flavonoid metabolites (**Supplementary Table S8**), including: *Rutin, Caffeoyl shikimic acid, Leucocyanidin, Sophoraflavonoloside, Troxerutin, 3,7-Di-O-methylquercetin, 3-O-Methylquercetin, Tricetin, Isoquercitrin, Quercetin*. It was observed that, except for gene *LOC107831218*, the expression levels of the other five genes regulating PAL and 4CL increase with nitrogen application. In the Flavonoid biosynthesis pathway, the expression of the genes HCT, CYP98A and CCoAOMT, except for *gene LOC107826706*, showed an increase with nitrogen. The metabolite *Caffeoyl shikimic acid*, on the other hand, showed a decrease with increasing nitrogen. Conversely, the gene expression levels of CHS, CHI, F3H, FLS, and CYP78B1 show a decreasing trend with increasing nitrogen levels. The metabolites *Troxerutin* and *Leucocyanidin* display a similar trend. In contrast, the expression of *Quercetin* expression increases, while the gene CYP78B1, which regulates the conversion of *Quercetin*, serves as a key gene connecting the Flavonoid biosynthesis and Flavone and flavonol biosynthesis pathways. In the Flavone and flavonol biosynthesis pathway, influenced by the up-stream genes and CYP78B1, the expression levels of *Quercetin, Isoquercitrin, Rutin, Sophoraflavonoloside, 3,7-Di-O-methylquercetin*, and *3-O-Methylquercetin* exhibit a regular pattern of increase and decrease with increasing nitrogen.

### 3.5 WGCNA analysis

**Figure 5:**
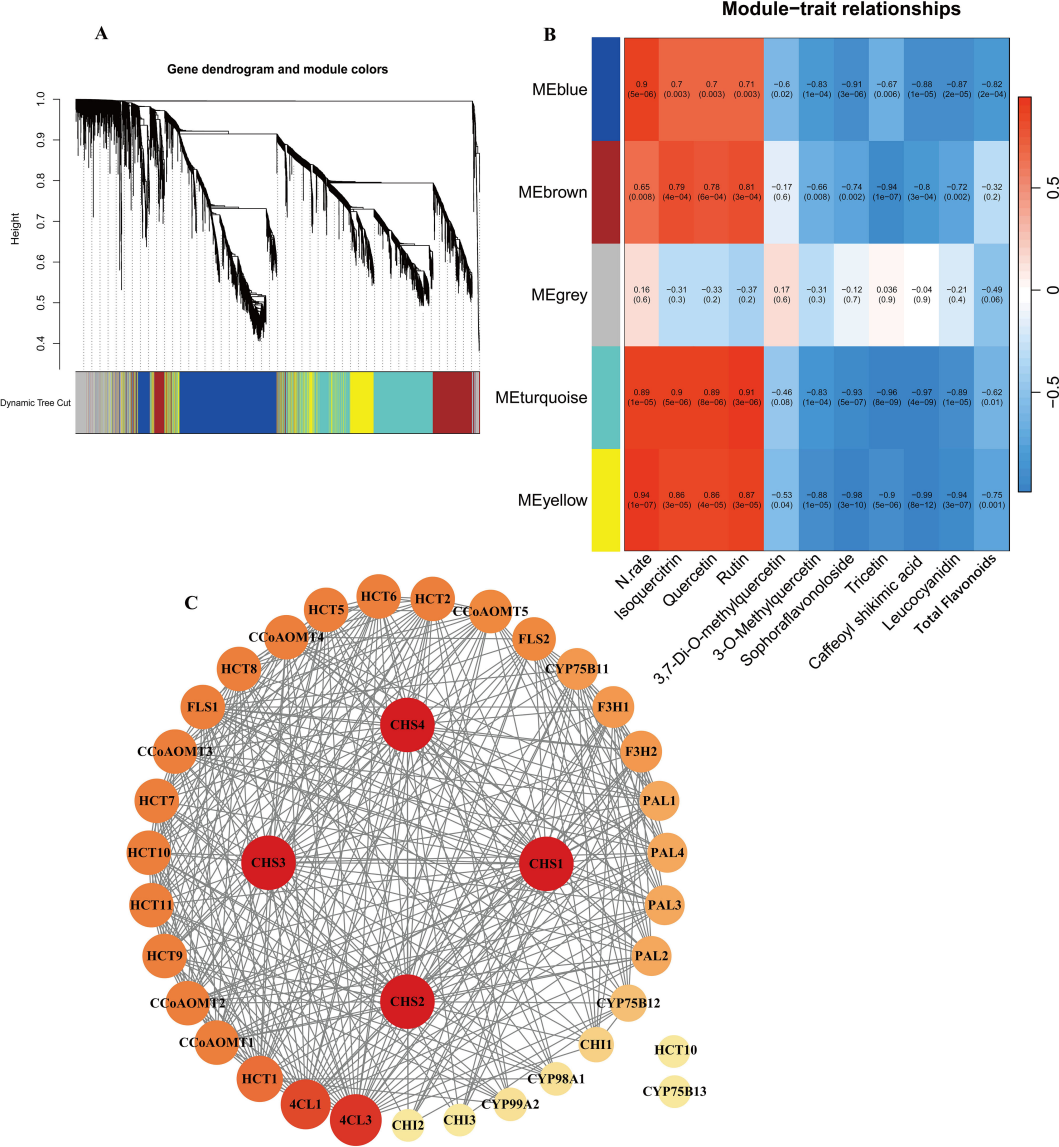
**(A)** Dendrogram from hierarchical clustering based on topological overlap. **(B)** Heatmap of correlations between module and sample traits. **(C)** PPI protein interaction analysis of candidate genes.

To further investigate and validate the regulatory process of genes involved in the flavonoid biosynthesis pathway, WGCNA was performed. A total of 69,782 genes detected in the samples were analyzed, filtering for genes with TPM greater than 1, resulting in 5,022 genes. The analysis used the nine DEMs annotated in the flavonoid biosynthesis pathway, nitrogen fertilizer rate (N.rate), and total flavonoid content as phenotypic data. A soft threshold of β = 14 was used, at which point the scale-free topological fit index *R*² was greater than 0.8 and the average connectivity converged to zero (**Supplementary Figure S2A**). The results suggested a significant similarity between the no nitrogen treatment (N0) and the low nitrogen treatment between the no nitrogen treatment (N0) and the low nitrogen treatment (N1), while the medium to high nitrogen treatments (N2, N3, N4) showed a higher degree of similarity (**Supplementary Figure S2B**). The results indicated the identification of five modules in the clustering dendrogram (**Figure 5A**), comprising 1,443 genes in the blue module, 798 genes in the brown module, 568 genes in the grey module, 1,470 genes in the turquoise module, and 743 genes in the yellow module (**Supplementary Table S9**). Notably, all modules, except for the grey module, showed significant correlation with nitrogen application levels. Additionally, four modules exhibited significant correlations with all metabolites total flavonoid content except for *3,7-Di-O-methylquercetin* (**Figure 5B**).

**Figure 6:**
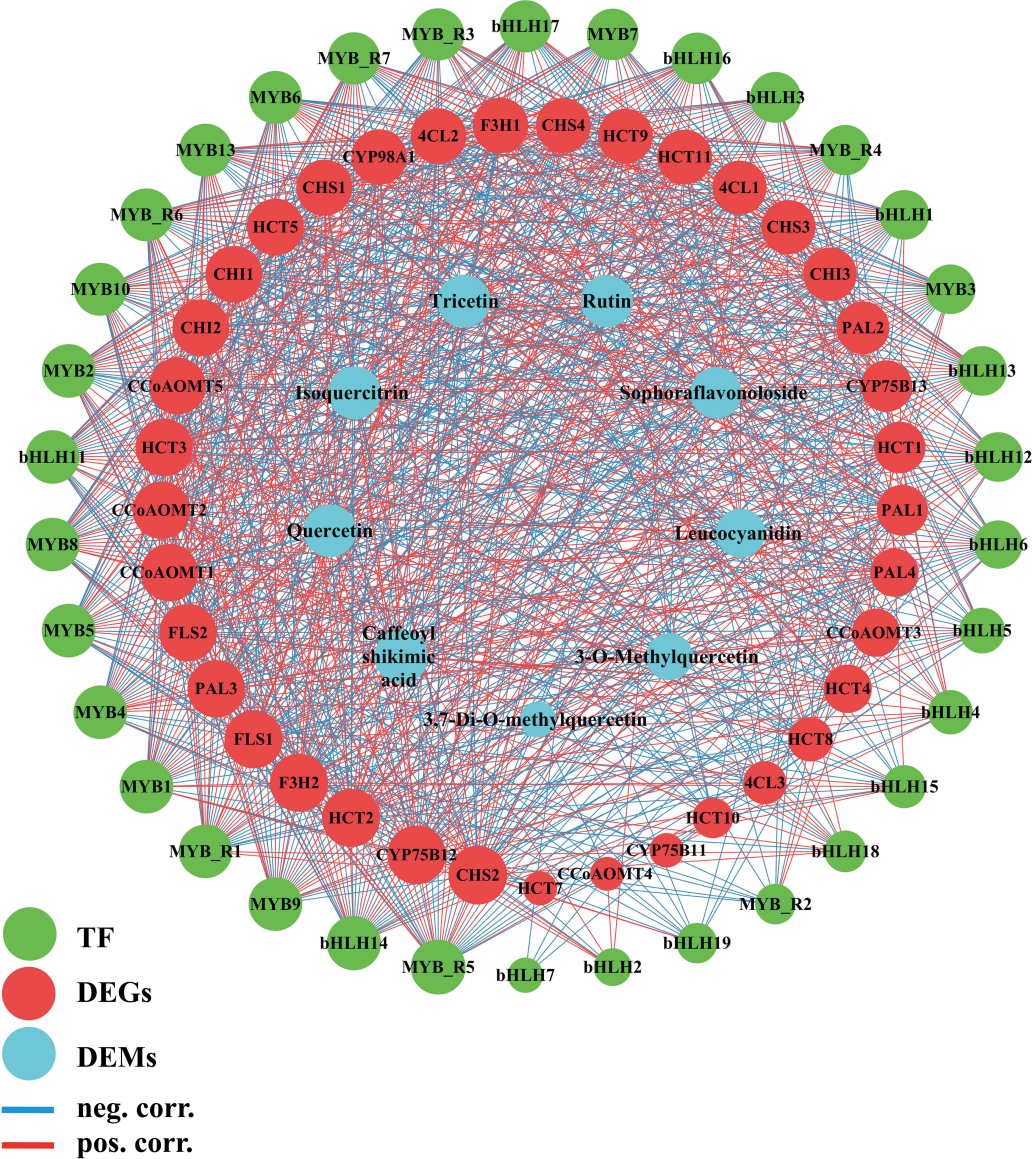
Transcription factor, genes, metabolite related co-expression network. The edges are drawn when the *p* value < 0.01. MYB_R stands for MYB_related.

KEGG enrichment analysis of these four modules (**Supplementary Figure S3, S4**) revealed distinct biological functions. The blue module was significantly enriched in fundamental plant metabolic pathways, including Plant-pathogen interaction, Plant hormone signal transduction, and Sesquiterpenoid and triterpenoid biosynthesis. The brown module was enriched in pathways such as Flavonoid biosynthesis, Isoquinoline alkaloid biosynthesis, and Pentose and glucuronate interconversions. The turquoise module showed significant enrichment in Arginine and proline metabolism, Photosynthesis antenna proteins, Phenylpropanoid biosynthesis, and Flavonoid biosynthesis. The yellow module was significantly enriched in Glutathione metabolism, Cyanoamino acid metabolism, and Alanine, aspartate, and Glutamate metabolism. Notably, both the brown and turquoise modules were significantly enriched in Phenylpropanoid and Flavonoid biosynthesis pathways. Therefore, we consider the brown and turquoise modules to be the core modules in this study. The genes in these modules were not only significantly enriched in the flavonoid biosynthesis pathways but also exhibited strong correlations with phenotypic data in the WGCNA analysis. To further screen for key genes in the flavonoid biosynthesis pathway, we selected a total of 39 genes, including genes from the brown and turquoise modules of the WGCNA results, as well as DEGs in the flavonoid biosynthesis pathway. Using the STRING database (https://cn.string-db.org/), with *Nicotiana tabacum* as the species, it was found that two of the inputted 39 genes had no proteins associated with that name in *Nicotiana tabacum*. Additionally, there are two outlier genes and two genes matching to corresponding proteins:*NtF3H-LOC107770893(F3H1)* (Zhang et al., 2021c) and *NtFLS-LOC107814657(FLS2)* (Wang et al., 2019) (**Supplementary Table S7**). The exported data was visualized using Cytoscape, resulting in a network with 37 nodes and 348 edges, demonstrating a dense protein-protein interaction network. To further investigate the critical genes associated with nitrogen response within flavonoid biosynthesis, we use the cytoNCA plugin to calculate degree centrality (DC) values and create a visual representation (**Figure 5C**). It was found that four *CHS* genes had the highest DC value of 32, followed by *4CL3*, *4CL1* and *HCT1, CCoAOMT1* and *CCoAOMT2*, which had a DC of 29, 27, 23, 21, 21. Thus, these genes are preliminarily identified as key regulators in the nitrogen response of flavonoid biosynthesis in cigar tobacco leaves. Flavonoid biosynthesis is primarily regulated by structural genes, while transcription factors mainly influence the synthesis of flavonoids by affecting these structural genes. Therefore, the role of transcription factors in flavonoid biosynthesis should not be underestimated. To clearly and intuitively display the relationships between potential transcription factors (genes), candidate genes, and flavonoid components outside the flavonoid biosynthesis pathway. Using the PlantTFDB database and focusing on the species *Nicotiana tabacum*, we identified a total of 104 genes containing 212 transcription factors, including ARF, B3, bHLH, bZIP, WRKY, MYB, and MIKC (**Supplementary Table S10**). We selected 20 bHLH and 20 MYB transcription factors (including 7 MYB-related factors) related to flavonoid biosynthesis (**Supplementary Table S11**). We analyzed these 40 transcription factors along with 39 core genes and their relationship with 9 metabolites. The analysis method employed was Pearson correlation analysis, using expression data and filtering for *p* value < 0.01 (**Supplementary Table S12**). Additionally, we utilized the cytoNCA plugin to calculate DC values (**Supplementary Table S13**) and arranged the genes according to their DC values (**Figure 6**).

The results indicate a highly significant relationship among transcription factors, regulated genes, and metabolite expression. To further identify core transcription factors and genes within this network, we filtered the data for *p <*0.01 and correlation coefficients > 0.95. In the relationship between genes and metabolites, there is a significant correlation between *PAL1, PAL2*, *CHS2, FLS2, CCoAOMT1, CCoAOMT5*, *Caffeoyl shikimic acid, Tricetin, Quercetin, Isoquercitrin, Rutin*. Although no correlation greater than 0.95 was found between *CCoAOMT2* and the metabolites, we discovered that it has significant correlations with nine metabolites in flavonoid biosynthesis. At the same time, *CCoAOMT1, CCoAOMT2, FLS2*, and *CHS2* also have multiple correlations with transcription factors. Additionally, in correlation network analysis of genes, transcription factors, metabolites, *CCoAOMT1, CCoAOMT2*, and *FLS2* have a DC value of 38, while *CHS2* has a DC value of 40, indicating that they are core genes linking transcription factors and metabolites. *CCoAOMT1, CCoAOMT2, FLS2*, and *CHS2* are also genes with high DC value in the protein interaction network results. In the relationship between transcription factors and structural genes, *MYB7, MYB8, MYB9, MYB10, bHLH14, MYB_related1*, and *MYB_related7* show significant correlations with *CCoAOMT1, CCoAOMT2, FLS2*, and *CHS2* (correlation > 0.77, *p* < 0.01). Therefore, we believe that *CCoAOMT1, CCoAOMT2, FLS2*, and *CHS2* are core genes in the nitrogen-regulated flavonoid biosynthesis pathway of cigar tobacco leaves, while *MYB7, MYB8, MYB9, MYB10, bHLH14, MYB_related1*, and *MYB_related7* are core transcription factors.

## 4 Discussion

Previous studies demonstrate that nitrogen supplementation enhances photosynthetic efficiency, stimulates amino acid biosynthesis, accelerates biomass accumulation, and optimizes agronomic performance in tobacco (Ripullone et al., 2003; Trápani et al.,1999). In this study, it is possible that the rapid plant growth with increased nitrogen fertilizer inputs led to leaf senescence, allowing for a decrease in SOD activity. Previous studies have also shown that leaf senescence leads to reduced SOD activity (Dhindsa et al., 1981). In the current study, the total flavonoid content decreased gradually with the increase in nitrogen fertilizer application. This observation is consistent with the general understanding that nitrogen reduces the accumulation of flavonoids in plants (Liu et al., 2010a; Nguyen and Niemeyer, 2008; Strissel et al., 2005). Flavonoids have antioxidant properties (D’Amelia et al., 2018), and SOD activity decreased with increasing nitrogen application, a trend consistent with changes in total flavonoid content. Previous research have also indicate that the total flavonoid content in tea was higher under no and low nitrogen treatments and lower under other nitrogen treatments (Liu et al., 2010a). This observation aligns with our findings, wherein total flavonoid content inversely correlated with N availability.

### 4.1 Structural genes and metabolites of flavonoid biosynthesis

Flavonoids are regulated by various structural genes involved in pathways such as phenylpropanoid, flavonoid, isoflavonoid, and flavonol biosynthesis (Borreda et al., 2022; Yi et al., 2022). This study identified two core modules related to the flavonoid biosynthesis pathway through WGCNA analysis and further screened the core genes *CHS2* and *FLS2* through protein-protein interaction network analysis. Both genes showed significant correlations with eight annotated metabolites and had high DC values in the protein interaction network. CHS is a key and rate-limiting enzyme in the flavonoid biosynthetic pathway (Deng et al., 2014; Zhang et al., 2017). In tomato (*Solanum lycopersicum*), the suppression of CHS mediated by RNA interference leads to a reduction in total flavonoid levels (Schijlen et al., 2007). Up-regulation of FLS can boost the metabolic flux towards flavonoid synthesis (Zhang et al., 2021b). In the current study, the expression of *CHS2* and *FLS2* was down-regulated under medium and high nitrogen treatments (N2, N3, and N4), and CHI and F3H showed a consistent expression trend between these two genes, which may be one of the reasons for the decrease in total flavonoid content. High nitrogen treatment reduces the activity of CHS and FLS enzymes, which in turn leads to a decrease in flavonoid content in apple leaves (Strissel et al., 2005), which aligns with the findings of this study.

In the analysis of metabolites, the levels of most metabolites showed a decreasing trend with increasing nitrogen application, consistent with the trend in total flavonoid content. The contents of *rutin*, *quercetin*, and *isoquercitrin* increased significantly with increasing nitrogen application, reaching the highest levels under high nitrogen treatment. Previous studies (Li et al., 2016; Yang et al., 2008) have shown that these compounds have strong antioxidant effects, and their accumulation may be a plant response to alleviate the oxidative stress induced under high nitrogen conditions. This was also corroborated with the increase in SOD activity with increasing nitrogen application. Lignin biosynthesis begins with the general phenylpropanoid pathway, which can also produce precursors for flavonoids. And the same substrates may be competed for by flavonoid synthesis and lignin biosynthesis (Zhang et al., 2021a; Zhu et al., 2019). A critical role of CCoAOMT in lignin deposition was evidenced by its enzymatic inhibition resulting in drastically decreased lignin content (Zhong et al., 2000). Meanwhile, PAL is also a key gene in lignin biosynthesis (Lu et al., 2019), and its expression trend is consistent with CCoAOMT. In this study, we discovered that nitrogen application promotes the expression of CCoAOMT. Therefore, we believe that the increase in CCoAOMT expression may enhance lignin synthesis, competing for precursor substances with flavonoids, which in turn leads to a decrease in total flavonoid content. The results of lignin content determination also support this viewpoint (**Supplementary Figure S5**). Plants may increase carbon flow to lignin synthesis by regulating carbon and nitrogen metabolism, thereby enhancing the mechanical strength of cell walls and improving stress resistance. This may be the reason why N0 treatment also has a higher lignin (Chen et al., 2018b).

### 4.2 Transcription factors in flavonoid biosynthesis

After constructing the flavonoid biosynthesis pathway map for cigar tobacco leaves, WGCNA analysis identified that MYB and bHLH are the most prevalent transcription factor families under different nitrogen treatments. Specifically, 20 MYB (including 7 MYB-related) transcription factors and 20 bHLH transcription factors were found to be associated with 37 structural genes involved in flavonoid biosynthesis. In addition, other transcription factor families such as ARF and B3 were also identified. When filtering for correlation > 0.95, *MYB7, MYB8, MYB9, MYB10, bHLH14*, and *MYB_related1, MYB_related7* were all significantly correlated with *CCoAOMT1, CCoAOMT2, FLS2*, and *CHS2* (correlations > 0.77). MYB and bHLH are two crucial transcription factor families in plants, and their interactions govern various enzymatic stages in flavonoid biosynthesis (Liu et al., 2021a; Totsuka et al., 2018; Xu et al., 2018). Previous studies have shown that the Arabidopsis transcription factor *AtMYB11* increases the content of beneficial flavonols in tobacco (Pandey et al., 2015). Additionally, the buckwheat gene *FtMYB31*, which encodes an R2R3-MYB transcription factor, promotes flavonoid accumulation in tobacco (Sun et al., 2020). We suggest that these transcription factors interact with each other to up-regulate the expression of *CCoAOMT1* and *CCoAOMT2* and repress the expression of *FLS2* and *CHS2* with increasing nitrogen application, which ultimately leads to a decrease in flavonoid content of tobacco leaves with increasing nitrogen application. Li’s study (Li et al., 2022) identified MYB as a prominent transcription factor family among those associated with nitrogen utilization. Furthermore, bHLH transcription factors have also been shown to enhance wheat’s tolerance to phosphorus and nitrogen deprivation (Yang et al., 2016). Therefore, we conclude that *MYB7, MYB8, MYB9, MYB10, bHLH14*, and *MYB_related1, MYB_related7* are key transcription factors responding to different nitrogen treatments in the flavonoid biosynthesis of tobacco.

In summary, the selected key genes and transcription factors are significantly correlated with the biosynthesis of flavonoids under nitrogen-responsive conditions. Nitrogen application may reduce the expression of *CHS2* and *FLS2* by affecting transcription factors, while upregulating the expression of *CCoAOMT1* and *CCoAOMT2*, thereby promoting lignin synthesis, which competes with flavonoid biosynthesis for common precursors, resulting in a decrease in total flavonoid content, which is consistent with our hypothesis. However, there were some differences between the qPCR results and transcriptome data for certain genes. Except for *CHS2*, the expression changes of *MYB7*, *MYB9*, *bHLH14*, and *MYB-related 1* observed in the transcriptome data were consistent with those in the qPCR results. Possible reasons may include sequencing bias, transcript isoform effects, or experimental technique errors. Future research should further explore how nitrogen application regulates transcription factors and associated metabolic pathways, further clarify the competitive relationship between lignin and flavonoid metabolism, and thus improve our understanding of the physiological mechanisms underlying nitrogen-regulated secondary metabolism.

## 5 Conclusions

By integrating transcriptomics and metabolomics, we constructed a regulatory network for flavonoid biosynthesis in cigar tobacco leaves and identified *CHS2* as a key gene, revealing the potential regulatory mechanism of nitrogen responsive flavonoid biosynthesis in cigar tobacco. Research has found that transcription factors *MYB7*, *MYB9*, *bHLH14*, and *MYB-related 1* play important roles in flavonoid biosynthesis. The nitrogen application treatment mainly reduced the expression of *CHS2* by regulating the expression of these transcription factors, affecting the accumulation of flavonoids and thus reducing the total flavonoid content. Our research provides a scientific basis for a deeper understanding of nitrogen-responsive flavonoid biosynthesis in cigar tobacco leaves, as well as important references for screening key genes related to nitrogen regulation and exploring plant growth regulatory networks.

## Data Availability

The datasets presented in this study can be found in online repositories. The names of the repository/repositories and accession number(s) can be found in the article/**Supplementary Material**.
