# Supplementary material for: Integrated transcriptomic and metabolomic analysis of flavonoid biosynthesis in cigar tobacco leaves under variable nitrogen regimes
Source: Front Plant Sci. 2025 Jun 23;16:1589215. doi: 10.3389/fpls.2025.1589215 (PMC12235917; doi:10.3389/fpls.2025.1589215)
Supplement: Supplementary file 2 [file Image1.pdf]

Supplementary Figures

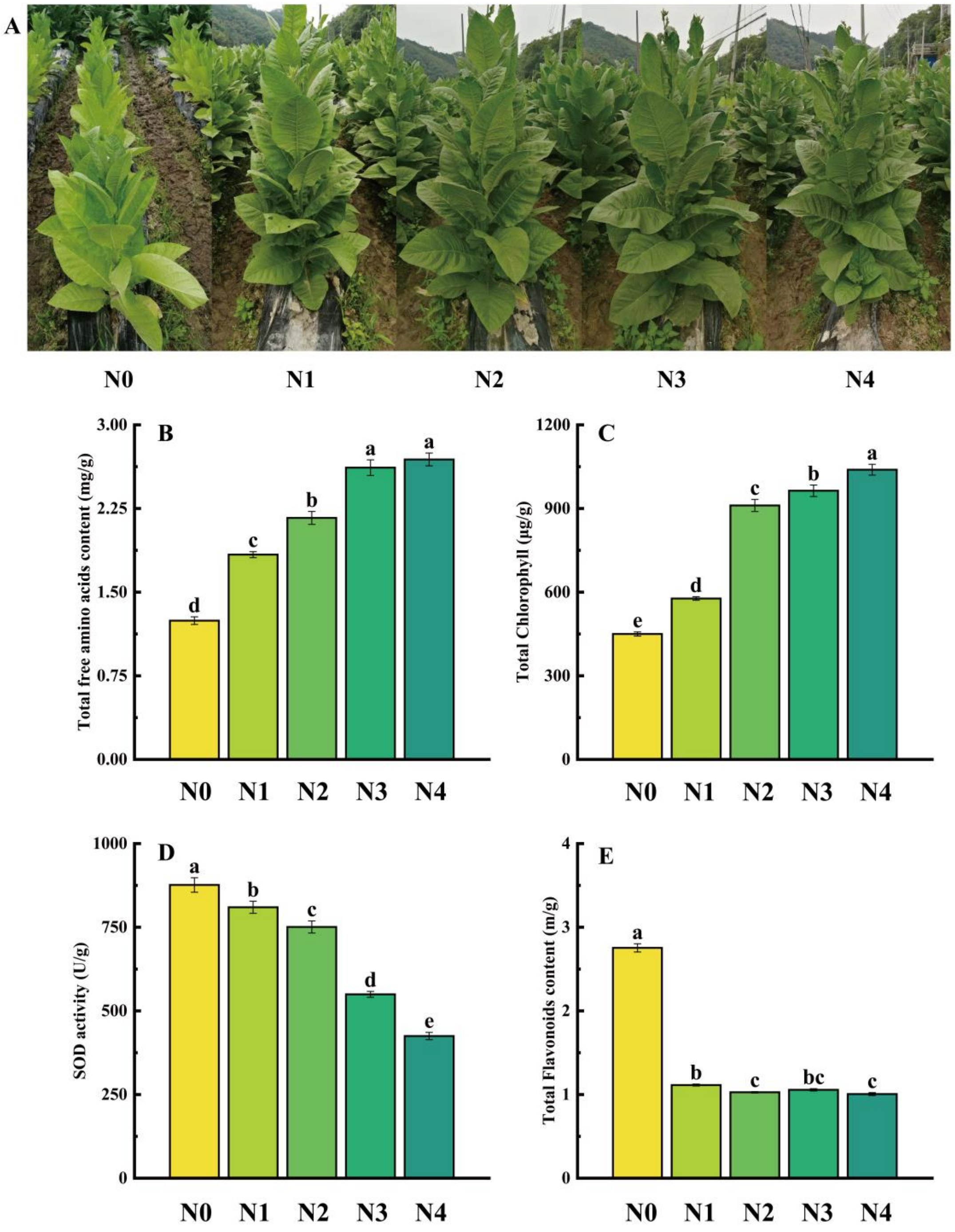

Fig. S1 **(A) The field maturity of cigar tobacco leaves. (B) Total free amino acid content. (C) Total chlorophyll content. (D) Superoxide dismutase (SOD) activity. (E) Total flavonoid content.**

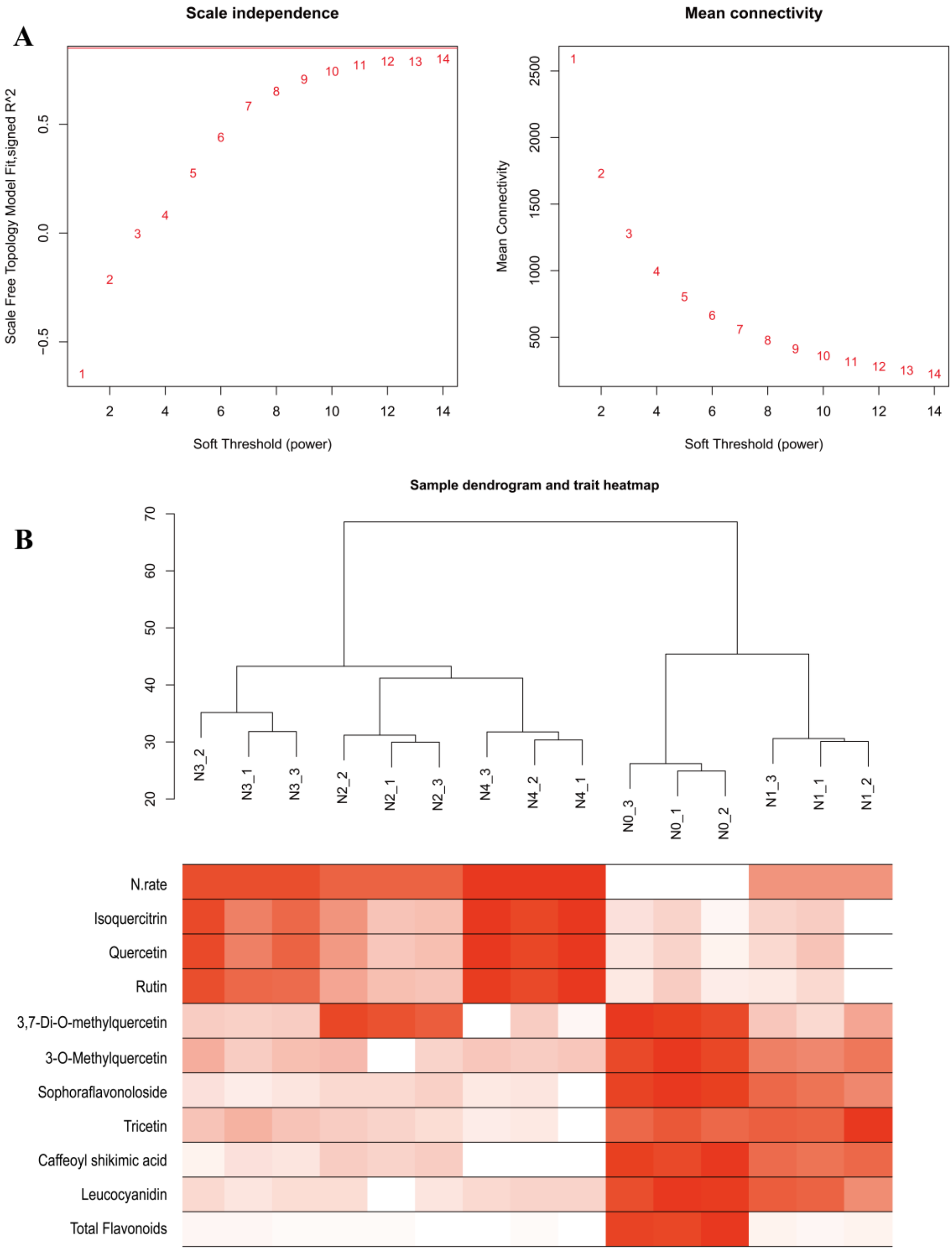

Fig. S2 (A) Scale independence and mean connectivity for WGCNA. (B) Sample dendrogram and trait heatmap.

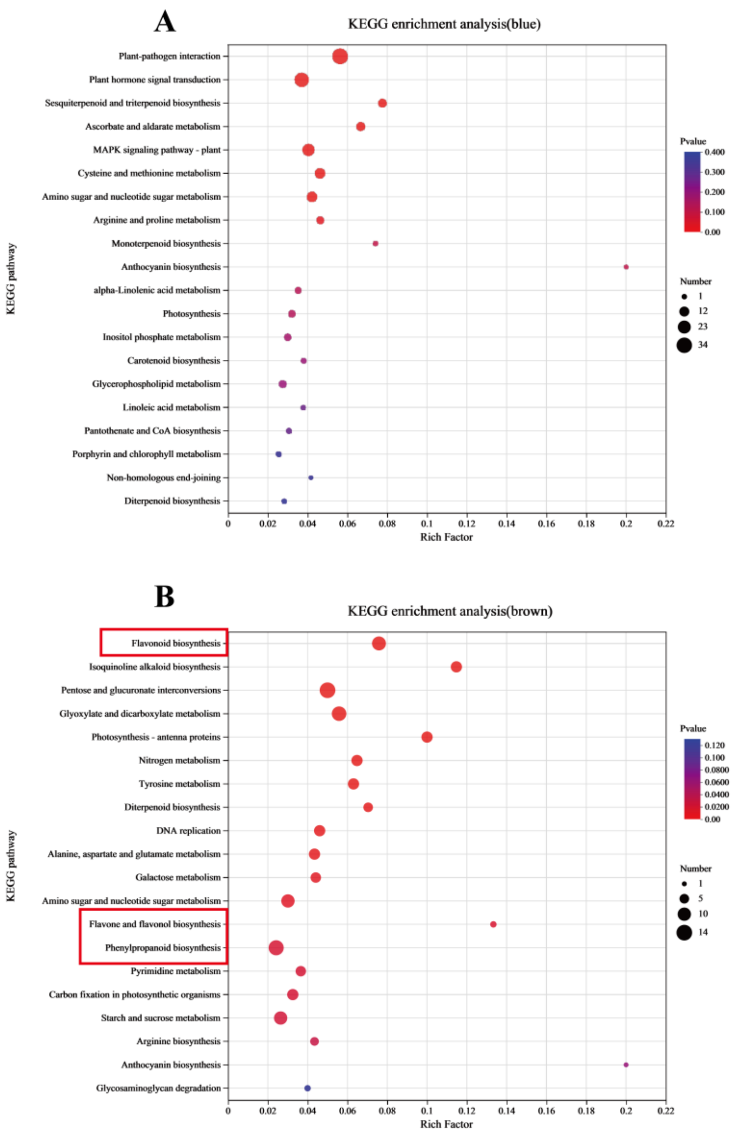

Fig. S3 Enrichment analysis graph of blue and brown modules. The red box represents pathways related to the biosynthesis of flavonoids ($p < 0.05$).

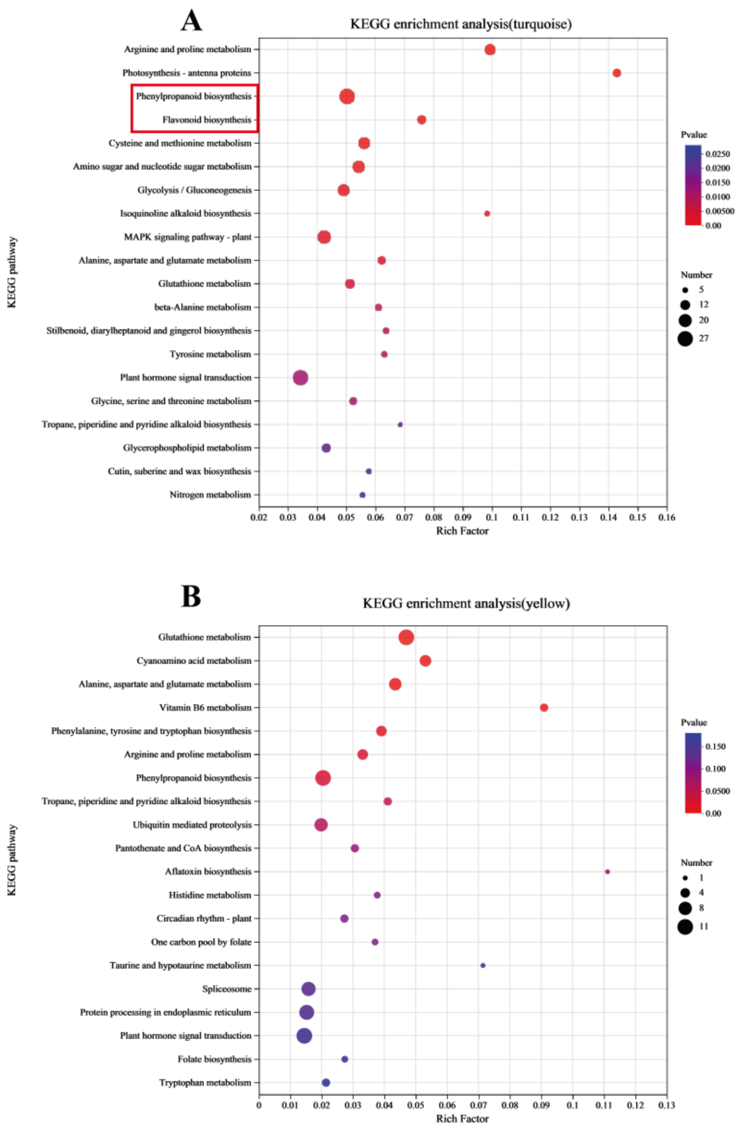

Fig. S4 Enrichment analysis graph of turquoise and yellow modules. The red box represents pathways related to the biosynthesis of flavonoids (p < 0.05).

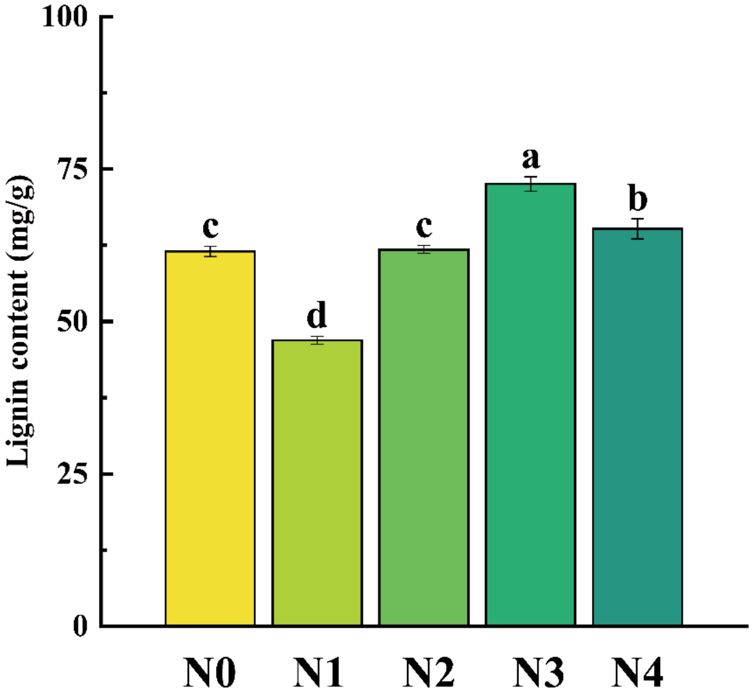

Fig. S5 Lignin content
